# Polyaniline Synthesized by Different Dopants for Fluorene Detection via Photoluminescence Spectroscopy

**DOI:** 10.3390/ma14237382

**Published:** 2021-12-02

**Authors:** Mahnoush Beygisangchin, Suraya Abdul Rashid, Suhaidi Shafie, Amir Reza Sadrolhosseini

**Affiliations:** 1 Nanomaterials Processing and Technology Laboratory, Institute of Nanoscience and Nanotechnology, University Putra Malaysia, Serdang 43400, Malaysia; m.beygi2300@yahoo.com; 2 Functional Nanotechnology Devices Laboratory, Institute of Nanoscience and Nanotechnology, University Putra Malaysia, Serdang 43400, Malaysia; 3 Faculty of Engineering, University Putra Malaysia, Serdang 43400, Malaysia; 4 Magneto-Plasmonic Laboratory, Laser and Plasma Research Institute, Shahid Beheshti University, Tehran 1983969411, Iran; amir17984818@gmail.com

**Keywords:** polyaniline, PANI, fluorene, dopant, PTSA, CSA, acetic acid, HCl, XRD, conductivity, four-point probe, sensor, photoluminescence

## Abstract

The effects of different dopants on the synthesis, optical, electrical and thermal features of polyaniline were investigated. Polyaniline (PANI) doped with p-toluene sulfonic acid (PANI-PTSA), camphor sulphonic acid (PANI-CSA), acetic acid (PANI-acetic acid) and hydrochloric acid (PANI-HCl) was synthesized through the oxidative chemical polymerization of aniline under acidic conditions at ambient temperature. Fourier transform infrared light, X-ray diffraction, UV-visible spectroscopy, field emission scanning electron microscopy, photoluminescence spectroscopy and electrical analysis were used to define physical and structural features, bandgap values, electrical conductivity and type and degree of doping, respectively. Tauc calculation reveals the optical band gaps of PANI-PTSA, PANI-CSA, PANI-acetic acid and PANI-HCl at 3.1, 3.5, 3.6 and 3.9 eV, respectively. With the increase in dopant size, crystallinity is reduced, and interchain separations and d-spacing are strengthened. The estimated conductivity values of PANI-PTSA, PANI-CSA, PANI-acetic acid and PANI-HCl are 3.84 × 10^1^, 2.92 × 10^1^, 2.50 × 10^−2^, and 2.44 × 10^−2^ S·cm^−1^, respectively. Particularly, PANI-PTSA shows high PL intensity because of its orderly arranged benzenoid and quinoid units. Owing to its excellent synthesis, low bandgap, high photoluminescence intensity and high electrical features, PANI-PTSA is a suitable candidate to improve PANI properties and electron provider for fluorene-detecting sensors with a linear range of 0.001–10 μM and detection limit of 0.26 nM.

## 1. Introduction

Fluorene is a polycyclic aromatic hydrocarbon (PAH) that poses risks to humans and the environment [1]. Several techniques have been developed for PAH detection, such as Fourier Transform Infrared spectroscopy, Raman spectroscopy, mass spectrometry (MS), gas chromatography (GC) and high-performance liquid chromatography [2]. Although these methods are sensitive and give reliable measurement, they are costly and require a long sample preparation time (non-real-time), bulky tabletop equipment and qualified operators. Others also suffer from low detection limits and necessitate a large amount of sample volume and solvent for separation and extraction.

Novel sensors for onsite fluorene detection, quantification and constant monitoring are vital to maintaining a healthy, non-polluted and sustainable environment. Technological growth in the field of sensors is driven by concerns about the safety and health of humanity [3]. Sensor devices are generally based on metal oxides operating at high temperatures. Hence, novel materials that can overcome the limitations of metal oxides are being explored.

Polymer systems provide a new avenue to synthesize novel materials with high thermal, electrical and optical features. Conducting polymers have remarkably attracted interest in the fields of nanoscience and nanotechnology due to their exceptional conductivity and redox performance [4]. A conductive polymer is a polymer that exhibits semiconductor or even conductor properties by chemically or electrochemically doping its backbone with a conjugated double bond [5,6]. The main conductive polymers, such as p-phenylenevinylene (PPV), polypyrrole (PPy) and polyaniline (PANI), are being applied in many areas, including photothermal therapy, electromagnetic interference shielding, photovoltaic cell, storage battery, membrane gas separation, microwave absorption, chemical sensors and anti-corrosion coating [7,8,9,10]. Some of their advantages include improved interface qualities, suitability for the production of lightweight devices, affordability and high productivity [10,11]. In polymerization, a plain organic synthesis technique must be applied to achieve a repeatable macromolecular design.

PANI is a singular conducting polymer compound with unique electrical and optical properties [10] and has the advantages of simple synthesis, environmental stability, affordability and flexible control of electrical features with charge-transfer doping and protonation [10,12,13]. Owing to its extraordinary properties, this compound can be used in various fields, such as electrochromic glasses [14], solar cells [15], electroluminescent machines [10], sensors [16], biosensors [17], supercapacitors [18], neural prosthesis/biotic–abiotic interfaces [10], scaffolding [19], delivery systems [20], anti-corrosion materials [21], membrane gas separation [8] and solar cells [22]. During the polymerization of aniline monomer, PANI transforms into one of the three states of normalized oxidation: (a) leucoemeraldine (white/clear), (b) pernigraniline (blue/violet) and (c) emeraldine (salt- green/base-blue). Pernigraniline is a completely oxidized PANI, emeraldine is half evenly oxidized and half decreased PANI, and leucomeraldine is completely decreased PANI [23]. The most conductive and consistent state is emeraldine with the conductivity of less than 10^−10^ s cm^−1^ created by its salt form. The synthesis method affects the conductivity of PANI, which can be controlled by immersing the emeraldine base in an aqueous acidic solution. PANI generally assumes a dark green solid-state after being formed through oxidative chemical polymerization inside an acidic aqueous average [10,24] in a solution of phosphoric acid, picric acid or camphor sulfonic acid [25,26,27,28].

**Figure 1:**
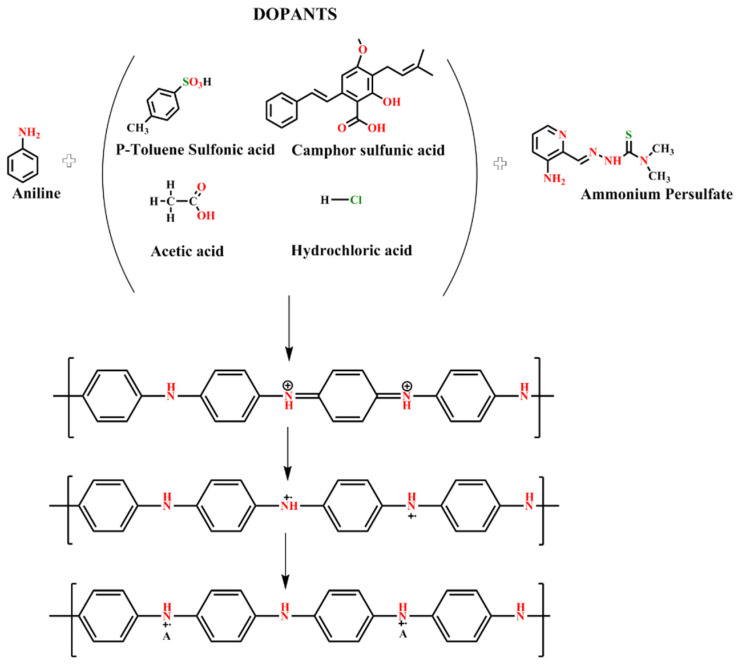
Synthesis of PANI by using different acids as dopants.

In the presence of an acid, PANI synthesis initially occurs through bipolar formation, followed by the development of a polar shape (Figure 1) [29,30,31,32,33]. The formation of a polaron structure leads to PANI-ES using protonation. Electrification of a conducting polymer causes the dopant to begin moving along the polymer; this phenomenon leads to the steady-state impairment because polarons can move along the polymer chains to enable electrical conductivity [34]. Doping either removes the electrons from the valence band molecular orbital or transfers them to the last empty transmission band molecular orbital. This process produces charge carriers, including polar and bipolar, inside the polymer [35,36]. The charge carriers of the polaron generate energy inside the dopant-restricted energy band to enable electron transfer from the valence to the conduction band.

PANI has a high potential to capture PAHs, such as fluorene that contains aromatic rings and a carboxyl functional group that can interact with PANI [37]. This compound also has promising chemical and physical properties such as high conductivity, environmental stability, corrosion resistance and homogenous microstructure. Additionally, PANI has functional groups such as sulfonated poly (aniline-co-o-aminophenol) and s-copolymer and can be prepared using oxidative chemical methods and doped with different acids [38]. Therefore, PANI is a suitable conductive polymer for PAH detection. Investigating the interaction of PANI with PAHs can help improve the fundamental understanding and application of PANI in sensors.

In this study, the effects of different acids as dopants on the physical properties and thermal, optical and electrical qualities of PANI were investigated. PANI was prepared through oxidative polymerization and doped using four types of acid, namely, organic acids such as toluene-4-sulfonic acid monohydrate (PTSA), camphor sulfonic acid (CSA) and acetic acid and inorganic acids such as hydrochloric acid (HCl). The prepared samples were examined by Fourier transform infrared (FT-IR) spectroscopy, X-ray diffraction (XRD), UV-visible spectroscopy (UV-vis), field emission scanning electron microscopy (FE-SEM), energy dispersive X-ray spectrometry (EDS), thermal gravity analysis (TGA) and photoluminescence (PL). The four-point probe method was used to investigate electrical behavior, and photoluminescence spectroscopy was applied to detect fluorescence properties. The application of PANI with excellent synthesis, features and high PL intensity to detect low fluorene concentration was also evaluated.

## 2. Materials and Methods

### 2.1. Materials

PTSA, aniline monomer (aniline 99%), CSA, hexane and fluorene were purchased from Merck KGaA, Darmstadt, Germany. *N*-Methyl-2-pyrrolidone, ammonium persulfate (APS 98%), acetic acid, HCl, ammonia solution (NH_4_OH) and H_2_O_2_ were provided by Avanti Chemicals of Merck KGaA, Darmstadt, Germany. Microscope glass slides were acquired from Jiangsu Huida Medical Instruments Co., Ltd. (Yancheng, Jiangsu, China). All chemicals were of analytical grade. Distilled water was used for aniline synthesis.

### 2.2. Preparation of PANI

PANI was synthesized as previously described [39]. Briefly, 2.75 mL of aniline was dissolved in 150 mL of 0.4 M PTSA, constantly stirred for 1 h, cooled and dropwise added with 25 mL of APS (1.7 g of APS dissolved in 25 mL of 0.4 M PTSA). The temperature was kept at 0–5 °C in an ice bath. The mixture was stirred for another 3 h. The precipitated raw polymer was filtered and washed using deionized water until it became colorless. The final substance was dehydrated in an oven at 50 °C for 12 h. In this form, PANI is known as emeraldine salt (ES). PANI was re-synthesized with three organic (CSA and acetic acid) and inorganic (HCl) acids (Table 1).

### 2.3. Preparation of Fluorene

PANI-PTSA with fluorene was prepared as follows. Briefly, 0.002 g of fluorene was dissolved in 250 mL of hexane containing 10 μM of fluorene solution to prepare aqueous fluorene solution. Other concentrations, i.e., 0.001, 0.01, 0.1, 1 and 10 μM were obtained by diluting the 10 μM fluorene solution as mentioned in Table 2. In all samples, fluorene amount was set as 160 μL.

### 2.4. Preparation of Thin Film

For the preparation of thin PANI layers, microscope glass slides were sonicated in ethanol for 10 min, then immersed in an aqueous solution of 4.0 mL of H_2_O_2_, 4.0 mL of NH_4_OH and 20.0 mL of deionized water for another 10 min, sonicated in deionized water and finally dried naturally. PANI solution with different dopants was adjusted to 80 μL. The thin film was deposited above a glass substrate by applying a spin coater (POLOS) at approximately 1500 rpm for 60 s. The remaining dispersions were stored in a safe place and monitored after 24 h.

### 2.5. Analytical Methods

FT-IR spectroscopy (Thermo Nicolet 6700 FT-IR) with KBr pellets was conducted in the range of 400–4000 cm^−1^ to categorize the functional groups and surface state of the PANI samples. X-ray diffraction (XRD) images were obtained by Shimadzu 6000 using Cu–Kα radiation at a scanning speed of 1.2° min ^−1^ of 3 to 90 degrees to ascertain the purity phase. Optical characteristics were determined by UV-vis spectroscopy (UV–vis, Lambda 35, PerkinElmer) at ambient temperature. Morphology was examined using a field emission scanning electron microscope (FE-SEM, NOVA NANOSEM 230). X-ray energy dispersive spectroscopy (EDS) was employed to determine the elemental composition of specific points or record the lateral distribution of elements in the imaged region. TGA analysis was adopted to study the thermal stability of the PANI samples below a nitrogen atmosphere from ambient temperature up to 900 °C at a heat range of 10 °C/min (Brand: METTLER TOLEDO). The photoluminescence excitation and emission wavelengths of 230 and 370 nm, respectively, were recorded in an aqueous solution utilising photoluminescence settings (PL, PerkinElmer, and LS 55). Electrical conductivity was measured using a four-terminal probe from Mitsubishi Chemical Analytech (MCCAT) Measuring Systems.

## 3. Result and Discussion

### 3.1. FT-IR Characterization

**Figure 2:**
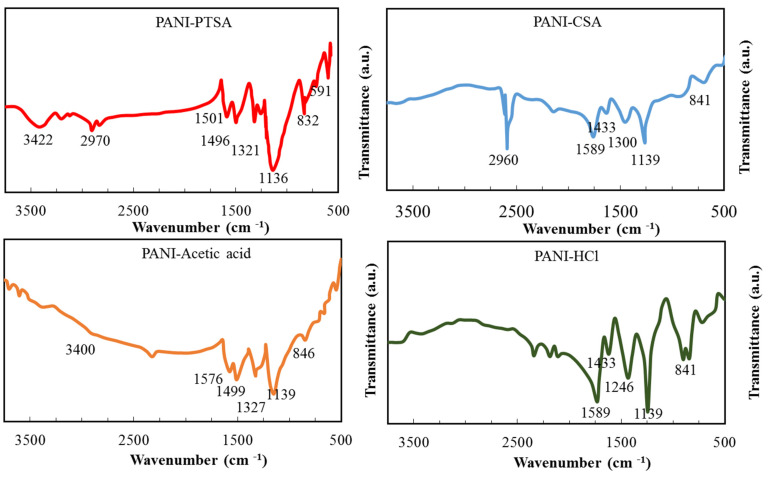
FT-IR spectra of synthesized PANI samples.

The components of PANI samples were examined by FT-IR analysis at 500–4000 cm^−1^. Figure 2 displays that doping with various acids effectively alters the PANI environment. All PANI samples are mainly of the same structure but still show significant differences from each other. The prominent vibrational bands shown in Table 3 are consistent with those previously reported for PANI salt [39].

The peaks for PANI-PTSA and PANI-HCl are sharper than those for PANI-CSA and PANI-acetic acid. Protonation converts PTSA and HCl to imine and amine nitrogen atoms, respectively, in PANI-PTSA and PANI-HCl structures, respectively. Benzenoid’s FT-IR absorption peaks and quinoid stretching fluctuations can predict the oxidation degree [39,40]. The peaks for PANI samples in Table 3 are consistent with those in previous reports [39,40,41,42].

In PANI-CSA and PANI-PTSA, the peaks at 2960 and 2970 cm^−1^ are associated with C-H stretching. These bands are not affected by doping, indicating that an aromatic ring is preserved inside the polymer [40].

### 3.2. XRD Characterization

**Figure 3:**
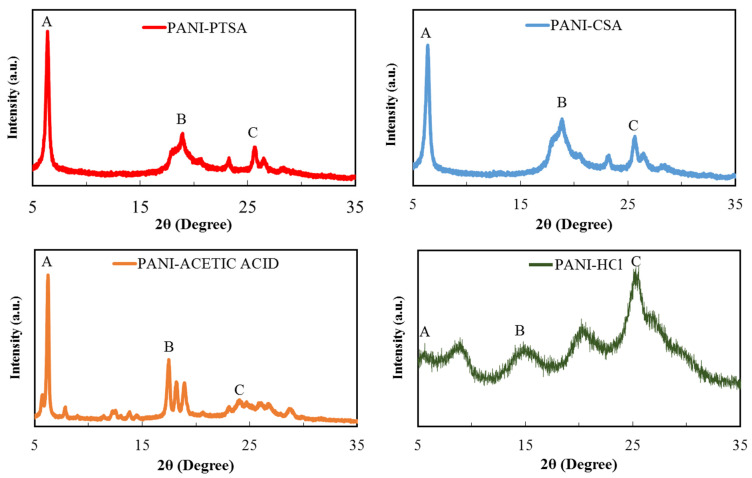
XRD spectra of synthesized PANI samples.

Figure 3 presents the XRD pattern of PANI doped with different acids, and Table 4 summarises their peak values. As indicated by differences in their diffraction patterns, the doping abilities of the dopants significantly vary under the same doping conditions. A broad diffraction pattern is obtained for all PANI samples due to their typical amorphous nature. XRD helps in estimating the size of inter-chain disconnection, crystallinity, crystallite size and d-spacing.

For all samples, d-spacing was delimited using Debye–Scherrer (powder) method utilizing the Bragg relation (Equation (1)) [43].

(1) nλ=2dsinθ,

where *n* is an integer; *λ* is the wavelength of X-ray, which for the Cu target is 1.54 Å; *d*, named as *d*-spacing, is the distance among the planes; and *θ* is the angle between the path of the beam and the planes.

Crystallite quantity was measured using the Scherrer relation (Equation (2)) [44];

(2) $$t=\frac{k\lambda }{B\;\cos\theta },$$

where *t* is the crystallite quantity, *K* is the ordinary crystallite (∼0.9) the shape factor and *B* is the entire diameter at crystalline peak’s half maximum in radians [45].

Inter-chain separation was determined using the relation given by Klug and Alexander (Equation (3)):

(3) $$R=\frac{5\lambda }{8\;\sin\theta },$$

where *R* is the inter-chain division.

The XRD profile for the PANI-PTSA pattern includes broad peaks at 6.4° and 18.97° and a sharp peak at 25.64°. The peak at 18.97° indicates the inter-chain distance between adjoining benzene rings in PANI, and the peak at 25.64° is attributed to the dispersion of PANI chains at an interplanar distance. On the basis of this microstructural analysis, PANI is in the verified form of ES [46]. PANI-CSA also shows similar peaks at 6.35° and 18.86° and a sharp peak at 25.61° [40]. PANI-acetic acid shows sharp, intense peaks at 6.26°, 17.49° and 24.72° attributed to the arrangement of the acetic acid molecules in the tunnels between the PANI chains [47]. Finally, PANI-HCl shows five peaks at 5.65°, 9.05°, 14.92°, 20.62° and 25.55° [48,49]. Given its long alkyl tails, PTSA can penetrate the crystal planes of PANI deeper than other acids with bulky cycloaliphatic rings such as CSA, acetic acid and HCl.

As shown in Table 4, all PANI samples have three crystalline peaks at 6°, 18° and 25°. Conductivity increases with the increasing crystallinity and decreasing inter-chain spacing and d-spacing. Table 5 describes the main properties of PANI samples obtained from the peak at 25° in the XRD characterization and their comparison with those from other research. A high grade of balance in the organization or order of polymer chains implies great crystallinity. Balance and ordered construction, defined as crystallinity, is a desirable factor, particularly for the intra-molecular movement of charged varieties towards the chain and to any degree for inter-molecular jumps due to dense and good packing. Therefore, an increase in crystallinity also increases conductivity. When the inter-chain spacing and d-spacing decrease, the possibility of inter-chain jumps increases, and conductivity simultaneously increases. Net conductivity is attributed to the intra-chain and inter-chain mobility of the electrons and holes [50,51]. Crystallinity reduction is in the order of PANI-PTSA > PANI-CSA > PANI-acetic acid > PANI-HCl. The actual quantity of inter-chain separation and d-spacing is the exact opposite of the theoretical quantity. Hence, conductivity decreases in the following order: PANI-PTSA > PANI-CSA > PANI-acetic acid > PANI-HCl. Nonetheless, the large acid exerts great force against the ordering and closing of polymer chains, resulting in low crystallinity, great inter-chain spacing and d-spacing and reduced conductivity.

### 3.3. UV-Vis Characterization

**Figure 4:**
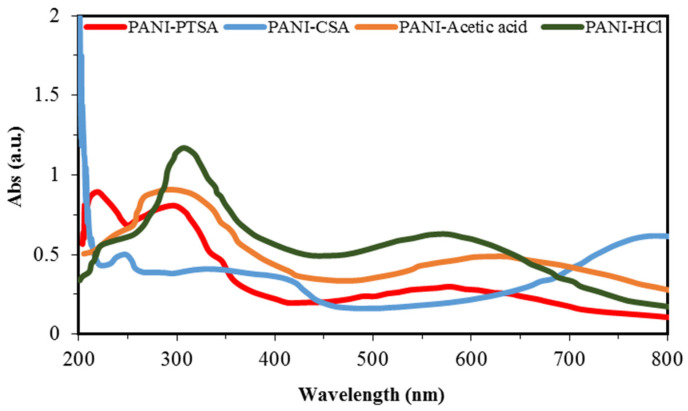
UV-vis spectra of synthesized PANI samples.

Figure 4 shows the UV-vis spectra of PANI samples. The absorption peaks of PANI samples depend on the doping level, conjugation extent and solvent type [57]. Valance and conduction bands are formed by the π and π* orbital, respectively.

Bandgaps are energy variations between two bands and are used to determine the optical properties of semiconducting polymers. Low bandgap energy implies smooth π–π* electronic transformation and increased conductivity [58]. The UV spectra of PANI samples exhibit three specific absorption bands across the wavelength regions of 250–320, 320–450 and 450–800 nm. Nitrogen excitation in the benzenoid segments (π–π* transition) causes the first absorption, and the shift from polaron to π* band and from π to polaron band of the doped PANI chains causes the second and third absorptions, respectively [53,58,59,60]. Table 6 shows the comparison of absorption rates from the UV-vis spectra of PANI samples. A decrease in absorption band at 578, 579 and 630 nm occurs for PANI-PTSA, PANI-HCl, and PANI-acetic acid, respectively. This phenomenon indicates that the quinoid rings and imine nitrogen atoms generated from protonation with PTSA, HCl and acetic acid dopants are transformed to benzenoid rings. The steric effect of the dopant anions may be the reason for the decrease in the peaks at 570 and 630 nm of the PANI samples [55].

Diffuse reflectance was analyzed to ascertain the energy range and the optical features of the PANI samples. Bandgap energies were calculated using the Tauc method, in which the energy-dependent absorption coefficient α is obtained using the following Equation (4):

(4) $${\left(\alpha h\upsilon \right)}^{2}=K\left(h\upsilon -Eg\right),$$

where *υ* is the frequency of the photon, *h* is Planck’s constant, *Eg* is the bandgap energy, *K* is a constant and *α* is the diffuse reflectance spectrum calculated from Equation (5):

(5) $$F\left(R\right)=\frac{1-R}{2R}.$$

**Figure 5:**
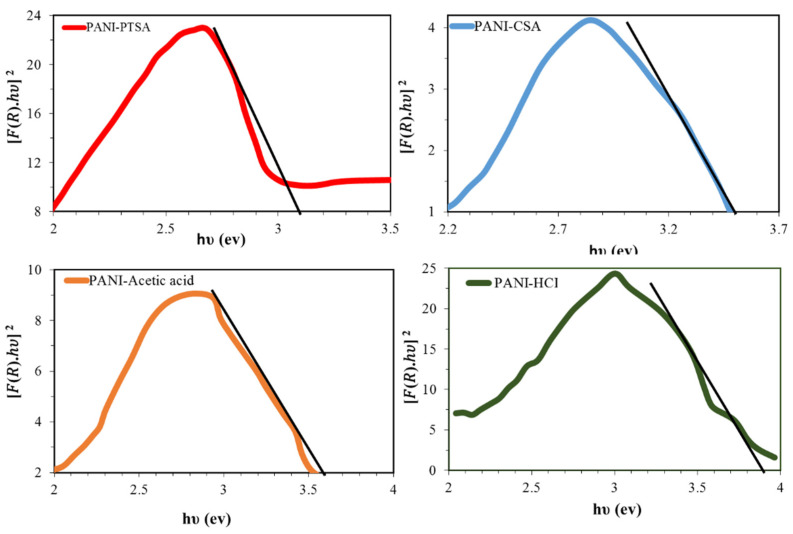
Bandgap curves as optical properties of the PANI samples.

Substituting *F*(*R*) in place of *α* in Equation (1) yields Equation (6):

(6) $${\left(F\left(R\right).h\upsilon \right)}^{2}=K\left(h\upsilon -Eg\right),$$

where *R* is the recognized reflectance inside UV-vis spectra, the crossing position within the [*F*(*R*)*. hυ*]^2^ versus *hυ* (Figure 5) implies that the bandgaps are an optical feature that can be calculated by extrapolating the straight line at *α* = 0. Therefore, the calculated optical bandgaps of PANI-PTSA, PANI-CSA, PANI-acetic acid and PANI-HCl were 3.1, 3.5, 3.6 and 3.9 eV, respectively. Laboratory experiment confirmed that the PANI bandgap differs significantly depending on the doping process. With increasing interaction between PANI and doping anions PTSA, CSA, acetic acid, and HCl and BSA, the conductivity of particles and the number of charge carriers increase, whereas the amount of bandgap decreases (Table 5).

### 3.4. FE-SEM and EDS Characterization

**Figure 6:**
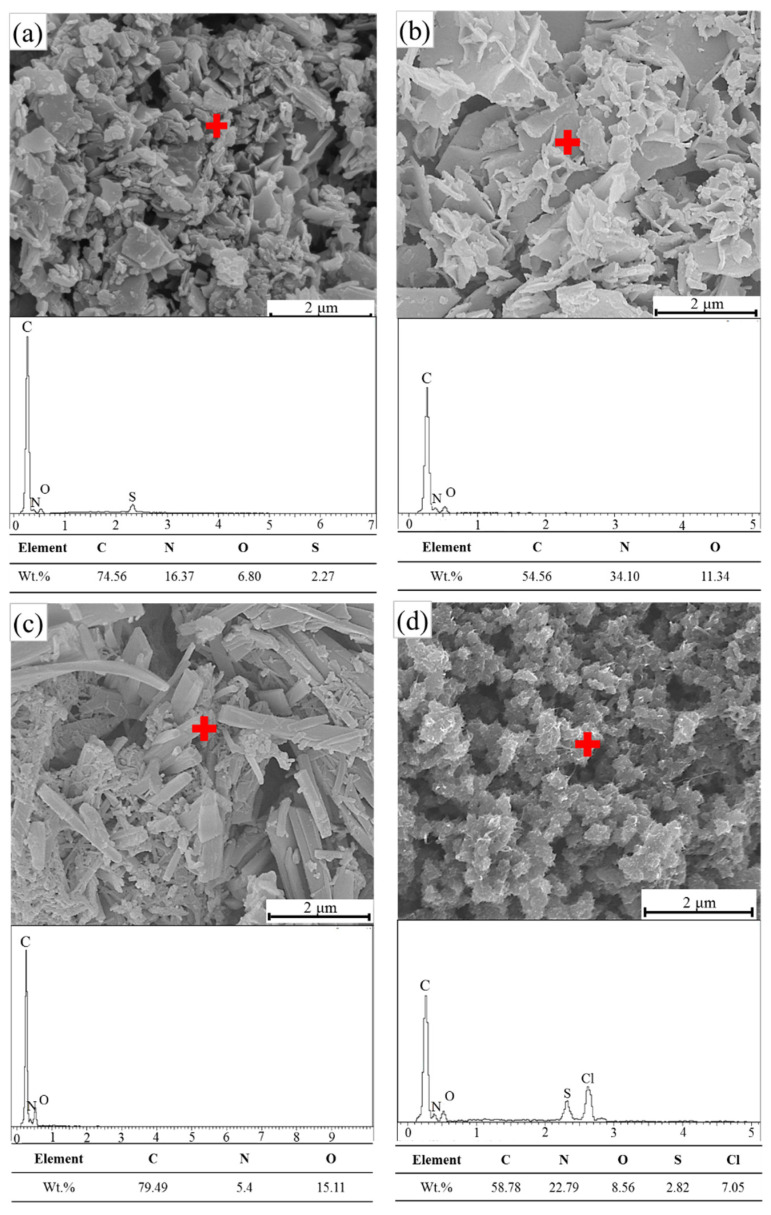
FE-SEM patterns and EDS point analysis of (**a**) PANI-PTSA, (**b**) PANI-CSA, (**c**) PANI-acetic acid and (**d**) PANI-HCl.

PANI morphology relies on the synthesis method, medium or solvent [61]. Figure 6 shows the FE-SEM images and elemental analysis of PANI samples prepared by using oxidative aniline polymerization techniques in organic and inorganic acid solutions. High surface tension is observed, leading to an aggregation tendency and a low specific surface area [62]. All the figures present the micrographs of PANI particles at a magnification of 10,000 with a scale bar of 2 μm. A granular structure (cluster) with micrometer-sized particles is found on the PANI surface, thus confirming the formation of ES [62]. By contrast, large aggregates of PANI particles with up to several microns in size are formed after drying as shown in FE-SEM images. Nanofibres are generally the fundamental units produced during the oxidative chemical polymerization of PANI [63,64,65]. Owing to secondary overgrowth, the generated final products become irregularly shaped PANI powders. Although the chemical polymerization of aniline can prevent secondary growth [66], aggregation remains crucial in this case. Agglomeration occurs due to the intra- and intermolecular ionic interchain interactions triggered by the proto-generated charge carriers (i.e., polarons/bipolarons) [67]. PANI-PTSA, PANI-CSA and PANI-acetic acid exhibit fibrous crystals when compressed. Meanwhile, PANI-HCl forms agglomerated ball crystals because the molecular chain stretches along the direction of mixing and shear forces during polymer crystallization [68].

The EDS results provide an explicit indication of the doped elements in the PANI samples. Chlorine (Cl) was generated from PANI doped by HCl. The importance of sulfur in the EDX pattern is because of using APS as an oxidant in polymerization.

### 3.5. TGA Characterization

**Figure 7:**
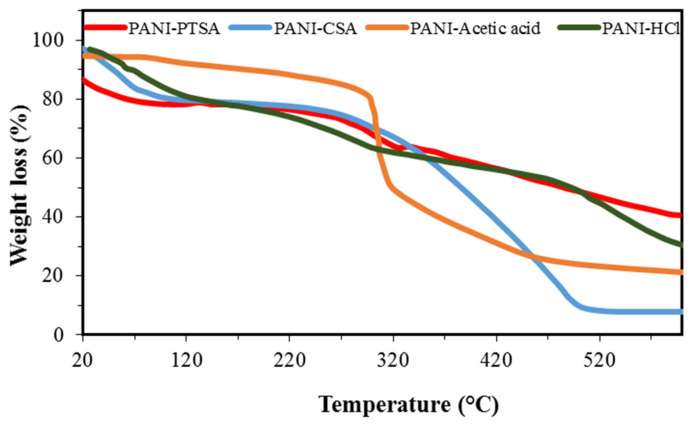
TGA spectra of synthesized PANI samples.

TGA was conducted to assess the thermal stability of the PANI synthesized in different acids, as shown in Figure 7, PANI-PTSA, PANI-CSA, PANI-acetic acid, and PANI-HCl. The mass losses were studied when heated in a nitrogen atmosphere. TGA curves for all samples show almost three regions of weight loss. In PANI-PTSA, the loss at around 108 °C is due to the elimination of moisture and other volatile compounds [69,70]. The second region starting at approximately 275 °C could be due to the loss of PTSA in the product [71]. The third region starting at approximately 391 °C could be due to the degradation of PTSA bonds [39].

Figure 7 shows that PANI-CSA and PANI-HCl exhibit almost three weight loss regions, particularly a weight loss at <120 °C caused by the evaporation or excretion of the imbibed water. Thus, moisture cannot be completely removed from the samples throughout the synthesis [72]. The second weight loss in PANI-HCl at 250 °C is due to the evaporation of HCl molecules. 

This intermediate, relatively small decomposition stage is discerned in PANI-CSA. The process begins at 286 °C and extends to 500 °C, after which a rapid decomposition occurs. Although the intermediate stage of degradation is highly evident with HCl doping at 490 °C, this phenomenon is also observed for PANI-CSA near ~502 °C, which represents the ultimate disintegration related to the polymer backbone. All the doped samples exhibit low disintegration temperature and low thermal resistance, except for the plasticized PANI-HCl with high estimated thermal stability [73].

Figure 7 shows the shape of the TGA thermograms for PANI-acetic acid. Solvent evaporation was conducted to purify the composites, and this procedure causes the first weight loss. Oligomer degradation causes the second weight loss (between 296 °C and 317 °C), and the evaporation of the sample causes the third weight loss of PANI–acetic acid [53].

PANI amount determines the thermal stability of the prepared composites. TGA results showed that acidity affects the thermal stability of PANI.

### 3.6. PL Analysis

**Figure 8:**
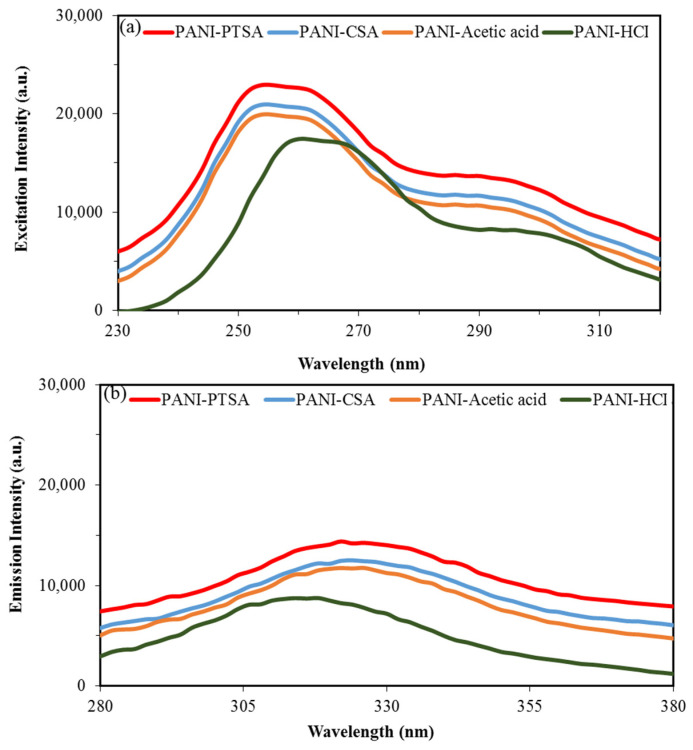
PL spectra of synthesized PANI samples. (**a**) Excitation photoluminescence peaks. (**b**) Emission photoluminescence peaks.

Figure 8 shows the measured PL spectra for the four samples. Excitation and emission wavelengths of 230 and 370 nm were chosen because the π–π* transition of benzenoid units is responsible for PL in PANI [74]. As shown in Figure 8a, the excitation peaks for all samples were observed at 258 nm, except for PANI-HCl with a peak at 264 nm. As shown in Figure 8b, the emission peaks for all samples were located at 326 nm, except for PANI-HCl with a peak at 316 nm. The PL intensity of PANI-HCl could be attributed to the appearance of few dopant ions (Cl−) in PANI-HCl that have greater charge mobility than the heavy dopant ions in other samples [73].

PANI-PTSA shows the highest PL intensity, followed by PANI-CSA, PANI-acetic acid and PANI-HCl. In PANI-PTSA, the benzenoid and quinoid parts are orderly arranged and thus support the formation of excitons and the enlargement of singlet exciton’s delocalization length [74,75]. Therefore, a high photoluminescence emission with a great extent of π conjugation should be expected from PANI-PTSA.

### 3.7. Electrical Analysis

A four-point probe was used to determine the conductivity of PANI samples at room temperature. The voltage difference can be observed when a current flows through the test specimen. Conversely, the resistance to current flow indicates conductivity.

Two parameters are crucial in PANI conductivity. One is the degree of oxidation, and the other is the degree of protonation [76]. The properties of PANI depend on the polymerization conditions (pH, temperature and time), monomer concentration, dopant type, the molar ratio of monomer to oxidant and solvent concentration [77]. PANI samples were prepared using different acids as dopants, and a four-point probe was applied to determine their electrical properties.

Resistance (Ω) was employed in accordance with the four-point probe technique. This parameter varies according to the nature, structure and composition of particles and the measurement position. Resistivity (Ω∙cm) is used to represent exact and actual substance characteristics and can be directly computed by multiplying the recorded resistance by a resistivity correction factor (RCF). Given that MCCAT resistivity measurement devices are designed to facilitate RCF computation, the resistivity was evaluated using a straightforward method. The sample size was set as 7.5 × 15 (mm) at the fixed position, and RCF was measured as 8.857.

In the four-point probe method, the resistance *R* (Ω), volume resistivity (*R_v_*) and surface resistivity (*Rs*) of samples were calculated using Equations (7)–(9):

(7) $$R\;\left(\Omega \right)=\frac{U\;\left(V\right)}{I\;\left(A\right)}.$$

According to the Ohm rule, the current (*I*) flowing through a conductor between two locations is equal to their potential difference (V) and inversely related to their resistance (*R*).

Volume resistivity (*R_v_*), also known as special resistance, is the resistance per unit volume of the specimen. This phrase is most commonly used in substance sorting (Ω∙cm). For this parameter, every substance is assumed to possess a particular specific percentage.

(8) $$R_{v}\left(\Omega .\mathrm{cm}\right)=R\;\left(\Omega \right)\times RCF\times t\;\left(\mathrm{cm}\right)$$

Surface resistivity (*R_s_*), commonly known as flake resistance, is the resistance per unit of the surface area of a specimen and is presented as Ω/sq to distinguish it from resistance. Given that *R_s_* fluctuates with the specimen diameter, most studies assess dye and fine coatings.

(9) $$R_{s}\left(\Omega ∕\mathrm{sq}\right)=R\;\left(\Omega \right)\times RCF=R_{v}\times t^{-1},$$

where *R* is resistivity (Ω), RCF is resistivity correction factor, and *t* [cm] is thickness. Thus, the effect of RCF on the resistivity of a specimen depends on measurement location, specimen dimensions and diameter.

Conductivity (*σ*), also called specific conductivity, is inversely related to volume resistivity (unit of S/cm or (Ω·cm)^−1^ and was computed using Equation (10). The obtained values are listed in Table 5 and Table 7.

(10) $$\sigma \;{\left(Ω.\mathrm{cm}\right)}^{-1}=\frac{1}{R_{v}}$$

Table 7 presents the electrical parameters of PANI samples. Their good conductivity values imply the successful production of adequate ES PANI type. The order of conductivity is PANI-PTSA > PANI-HCl > PANI-CSA > PANI-acetic acid. Intensity and charge carrier mobility determine the net conductivity of doped PANI and depend on the dopant nature, the structure of the doped PANI and preparation method [50,51,58,78].

### 3.8. Fluorescence Detection of Fluorene

**Figure 9:**
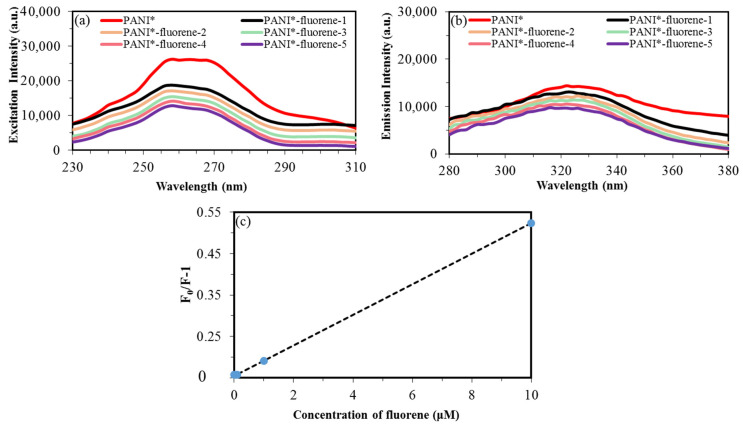
(**a**) Excitation photoluminescence peaks. (**b**) Emission photoluminescence peaks of fluorene in concentrations of 0.001, 0.01, 0.1, 1 and 10 μM upon interaction with PANI*. (**c**) Variation of intensity peaks when PANI* interacted with fluorene.

PL studies confirmed that PANI-PTSA has the best PL intensity among the samples. This finding could be attributed to the orderly arrangement of benzenoid and quinoid units which support the formation of excitons and the enlargement of singlet exciton’ delocalization length. With its advanced PL and electrical properties, PANI-PTSA (PANI*) shows an excellent crossing point of providing electrons in sensor application and therefore was selected for fluorene detection. Figure 9 depicts the PL intensity when PANI-PTSA was used detect various fluorene concentrations (0.001, 0.01, 0.1,1 and 10 μM). As shown in Figure 9a, the specific excitation intensity that activates photoluminescence when PANI* is in contact with fluorene appears at 258 nm. As displayed in Figure 9b, the photoluminescence emission peak related to PANI* in contact with fluorene appears at 324 nm. These results indicate that when fluorene concentration increases from 0.001 μM to 10 μM, the intensity of the photoluminescence peak decreases [79,80].

Stern–Volmer equation was used to study the interaction between PANI and fluorene (Equation (11)) as follows:

(11) $$\frac{F_{0\;}}{F}-1=K_{sv}\times C_{DA,}$$

where *F* and *F*_0_ are the PL intensity of PANI*-fluorene systems and PANI* solution, respectively; CDA is the quencher (fluorene) concentration, and *K_sv_* is the Stern–Volmer quenching constant [81].

A good linear relationship is obtained between PL intensity ratio (F_0_/F-1) and fluorene concentration in the range of 0.001–10 μM (Figure 9c). A photoluminescence test was carried out 10 times to evaluate the stability of the photoluminescence properties of PANI*. The standard error, standard deviation (*σ*) and slope of variation curve are 0.0009, 0.002 and 0.026, respectively. The limit of detection (LOD) is proportional to the standard deviation (*σ*) and slop (*K*) of variation of photoluminescence intensity with fluorene concentration. Therefore, the LOD (LOD = (3.3 × *σ*)/*K*) is approximately 0.26. Table 8 shows the comparison of detection results for fluorene sensors with different fluorescence spectroscopy methods.

## 4. Conclusions

PANI-PTSA, PANI-CSA, PANI-acetic acid and PANI-HCl were successfully prepared through chemical oxidative polymerization. The optical, thermal, and electrical properties of PANI rely on the nature and particle quantity of the acid dopant. UV-vis and FT-IR spectra showed that doping creates fundamental varieties inside PANI chains. PANI-PTSA, PANI-CSA and PANI-acetic acid appear as fibrous crystals when compressed. PANI–HCl presents as agglomerated ball crystals because the molecular chain stretches along the direction of mixing and shear forces during polymer crystallization. An XRD study revealed that the chain length grows with the increasing acidity. When the dopant quantity increases, crystallinity decreases, and inter-chain separation and d-spacing increase. As a result, the mobility of load carriers along with a polymer chain and over chains decreases, and conductivity declines. PANI-PTSA exhibits the highest conductivity among the PANI samples. Additionally, TGA results implied that acidity affects the thermal stability of PANI. PL studies confirmed that among the samples, PANI-PTSA has the best PL intensity because of the orderly arrangement of benzenoid and quinoid units that support the formation of excitons and the enlargement of singlet exciton’ delocalization length. Therefore, PANI-PTSA (PANI*) with advanced PL and 0.26 nM detection limit shows a good balance of providing an electron and detecting fluorene in sensor applications.

## Figures and Tables

**Table 1:** Preparation of PANI samples.

Sample Name	Aniline Monomer Concentration (mL)	APS as Oxidant Monomer (g)	Different Acids (0.4 M)	Weight of the PANI (g)
PANI-PTSA	2.75	1.7	PTSA	0.2
PANI-CSA	2.75	1.7	CSA	0.18
PANI-Acetic acid	2.75	1.7	Acetic acid	0.15
PANI-HCl	2.75	1.7	HCl	0.19

**Table 2:** Preparation of PANI-PTSA with fluorene concentrations.

Sample Name	Amount of PANI* (μL)	Amount of Fluorene (μL)	Fluorene Concentrations (μM)
PANI-PTSA-fluorene-1	2900	100	0.001
PANI-PTSA-fluorene-2	2900	100	0.01
PANI-PTSA-fluorene-3	2900	100	0.1
PANI-PTSA-fluorene-4	2900	100	1
PANI-PTSA-fluorene-5	2900	100	10

**Table 3:** Comparison of functional group peaks in the FT-IR spectra of the PANI samples.

Assignment		PANI-PTSA [39]	PANI-CSA [40]	PANI-Acetic Acid [41,42]	PANI-HCl [40]
Symmetric and asymmetric stretching	NH_2_ and NH	3422	-	3400	-
Aromatic aniline ring	C–H and CH_2_	2970	2960	-	-
Quinoid ring stretching	C = C	1501	1589	1576	1589
Benzonoid ring stretching	N–B–N	1496	1433	1499	1433
C-N stretching of benzenoid ring	C–N	1321	1300	1327	1246
In-plane bending vibration of C-H	C–H	1136	1139	1139	1139
Ortho substitutions, 1,2 disubstitution in benzene ring	C–H	832,591	841	846	841

**Table 4:** Crystallite size, d-spacing and interchain division of PANI samples exposed over various shots of ES reflection (measured from XRD).

Sample Name	Peak Sign	d-Spacing (Å)	FWHM (B)	Crystallite Size (nm)	Inter-Chain Separation (Å)
PANI-PTSA-1	A	15.71429	0.006	241.750167	19.64285714
PANI-PTSA-2	B	4.695122	0.044	33.36713996	5.868902439
PANI-PTSA-3	C	3.484163	0.017	87.33634992	4.35520362
PANI-CSA-1	A	14.25926	0.008	181.3126253	17.82407407
PANI-CSA-2	B	4.723926	0.051	28.78733644	5.904907975
PANI-CSA-3	C	3.494163	0.027	59.56613339	4.36520362
PANI-Acetic acid-1	A	14.5283	0.004	362.6252505	18.16037736
PANI-ACETIC acid-2	B	5.099338	0.02	72.5250501	6.374172185
PANI-ACETIC acid-3	C	3.615023	0.0249	54.98955366	4.375
PANI-HCl-1	A	16.04167	0.037	39.20272978	20.05208333
PANI-HCl-3	B	5.968992	0.091	16.05216176	7.46124031
PANI-HCl-5	C	3.5	0.107	13.87586868	4.518779343

**Table 5:** Features of synthesized PANI samples and comparison with other research.

Sample Name	Crystallite Size (nm)	d-Spacing (Å)	Inter-Chain Separation (A˚)	Bandgap	Conductivity (S·cm^−1^)	References
PANI-PTSA	47	3.50	4.37	4.50	4.8 × 10^−2^	[30]
	13.8	3.51	4.39	4.196	1 × 10^−2^	[50]
	29	4.6	-	-	12 × 10^−2^	[46]
	87	3.48	4.35	3.1	3.84 × 10^1^	Current study
PANI-CSA	-	3.52	-	3.8	11 × 10^−2^	[52]
	65	4.9	-	-	21 × 10^−2^	[46]
	56	-	-	-	2.7× 10^1^	[40]
	59	3.49	4.36	3.5	2.92 × 10^1^	Current study
PANI-Acetic acid	49	-	-	-	6.5× 10^−2^	[53]
	-	-	-	-	4.21 × 10^−2^	[32]
	-	-	-	-	6.5 × 10^−5^	[54]
	55	3.6	4.37	3.6	2.50 × 10^−2^	Current study
PANI-HCl	59	3.49	4.36	4.48	1.6 × 10^−1^	[30]
	-	-	-	2.40	54 × 10^−2^	[55]
	38.3	3.9	-	3.85	-	[56]
	14	3.5	4.5	3.9	2.44 × 10^−2^	Current study

**Table 6:** Comparison of the absorbance values of the PANI samples.

Π–π*		Polaron to π*		π to Polaron	
PANI-Samples	Wavelength (nm)	PANI-Samples	Wavelength (nm)	PANI-Samples	Wavelength (nm)
PANI-PTSA	207	PANI-PTSA	321	PANI-PTSA	578
PANI-CSA	253	PANI-CSA	338	PANI-CSA	785
PANI-Acetic acid	283	PANI-Acetic acid	-	PANI-Acetic acid	630
PANI-HCl	317	PANI-HCl	-	PANI-HCl	579

**Table 7:** Electrical data of PANI samples.

Sample Name	*R* (Ω)	*R_s_* (Ω/sq)	*R_v_* (Ω·cm)	Conductivity (Ω·cm)^–1^
PANI-PTSA	9.98 × 10^−1^	8.84	2.61 × 10^−2^	3.84 × 10^1^
PANI-CSA	1.31 × 10^−1^	1.16	3.42 × 10^−2^	2.92 × 10^1^
PANI-Acetic acid	1.53 × 10^3^	1.36 × 10^4^	4.00 × 10^−2^	2.50 × 10^−2^
PANI-HCl	1.57 × 10^3^	1.39 × 10^4^	4.10 × 10^1^	2.44 × 10^−2^

**Table 8:** Comparison of fluorene sensors with different detection techniques according to fluorescence obtained during experimental measurements.

No.	Fluorescent Sensor Methods	Linear Range (μM)	LOD (nM)	References
1	Eu(III)-(CCA)_2_ probe	0.1–1.9	0.43	[82]
2	Thin-layer chromatography (TLC)	-	0.5	[83]
3	1,3-bis(cyanomethoxy)-tert-butylcalix[4]arene (CAD) onto CdSe quantum dots (QDs) CAD@CdSe	0–20	0.8	[84]
4	PANI doped PTSA	0.001–10	0.26	Current study

## Data Availability

The data presented in this study are available on request from the corresponding author.
